# Life-history tradeoffs in a historical population (1896–1939) undergoing rapid fertility decline: Costs of reproduction?

**DOI:** 10.1017/ehs.2022.2

**Published:** 2022-02-21

**Authors:** Adrian V. Jaeggi, Jordan S. Martin, Joël Floris, Nicole Bender, Martin Haeusler, Rebecca Sear, Kaspar Staub

**Affiliations:** 1 Institute of Evolutionary Medicine, University of Zurich, Zurich, Switzerland; 2 Department of History, University of Zurich, Zurich, Switzerland; 3 London School of Hygiene and Tropical Medicine, London, UK; 4 Zurich Center for Integrative Human Physiology, University of Zurich, Zurich, Switzerland; 5 Institute of History, University of Bern, Bern, Switzerland

**Keywords:** Demographic transition, quantity–quality tradeoff, life-history theory, evolutionary demography, historical demography

## Abstract

Evolutionary demographers often invoke tradeoffs between reproduction and survival to explain reductions in fertility during demographic transitions. The evidence for such tradeoffs in humans has been mixed, partly because tradeoffs may be masked by individual differences in quality or access to resources. Unmasking tradeoffs despite such phenotypic correlations requires sophisticated statistical analyses that account for endogeneity among variables and individual differences in access to resources. Here we tested for costs of reproduction using *N =* 13,663 birth records from the maternity hospital in Basel, Switzerland, 1896–1939, a period characterised by rapid fertility declines. We predicted that higher parity is associated with worse maternal and offspring condition at the time of birth, adjusting for age and a variety of covariates. We used Bayesian multivariate, multilevel models to simultaneously analyse multiple related outcomes while accounting for endogeneity, appropriately modelling non-linear effects, dealing with hierarchical data structures, and effectively imputing missing data. Despite all these efforts, we found virtually no evidence for costs of reproduction. Instead, women with better access to resources had fewer children. Barring limitations of the data, these results are consistent with demographic transitions reflecting women's investment in their own embodied capital and/or the adoption of maladaptive low-fertility norms by elites.

## Graphical abstract

**Figure:**
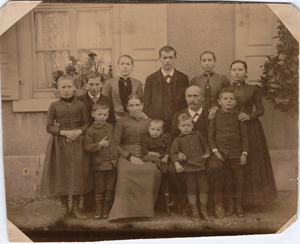

**Social media summary:** Why did they have fewer babies? Registered report finds no quantity–quality tradeoffs in historical Basel (1896–1939).

## Introduction

Evolutionary demographers are interested in explaining variation in fertility, mortality and parental investment across populations (Lawson & Borgerhoff Mulder, 2016; Mace, 2000; Sear, Lawson, Kaplan, & Shenk, 2016). In doing so, they often make use of life-history theory (LHT), which was developed in evolutionary biology to explain such variation across species (Charnov, 1991; Charnov & Berrigan, 2005; Roff, 1993; Stearns & Koella, 1986; Stearns, 1992). Empirical tests of LHT in humans are important as deviations from predictions may highlight species-typical patterns not accounted for by the standard theory, such as the importance of allomaternal investment, mutual mate choice or embodied capital (Blurton Jones, 2016; Gurven et al., 2016; Hill & Hurtado, 1996; Lawson & Borgerhoff Mulder, 2016; Shenk, Kaplan, & Hooper, 2016). However, most evolutionary demography has focused on pre- or post-demographic transition societies, such as hunter–gatherers (Blurton Jones, 2016; Hill & Hurtado, 1996) or present-day industrialised societies (Nettle et al., 2013; Uggla & Mace, 2015), as well as populations in developing countries experiencing the early stages of the demographic transition (Alvergne & Lummaa, 2014; Lawson, Alvergne, & Gibson, 2012; Sear, Mace, & McGregor, 2003). Here we test for costs of reproduction in the rarely studied context of an urban, industrialised population during a 40+ year period of rapid fertility decline, representing mid- to late stages of the demographic transition.

Fundamental to LHT is the concept of tradeoffs (Charnov, 1991; Stearns, 1989), wherein finite time or energy allocated to one functional domain, such as growth, reproduction or survival, is no longer available to others. A fundamental life history tradeoff occurs between current reproduction and future survival and reproduction. Thus, larger offspring or litter size and shorter spacing of reproductive events may come at a potential cost of reduced future reproductive potential and/or survival for the mother (the so-called ‘cost of reproduction’), as well as a potential cost for the offspring (‘quantity–quality tradeoff’ or ‘intergenerational costs of reproduction’; Charnov, 1991; Stearns, 1989). As such, higher fertility and/or parental investment should lead to reduced maternal health and longevity, for which there is good evidence from various species (Healy et al., 2019; Knowles, Nakagawa, & Sheldon, 2009; Santos & Nakagawa, 2012; Stearns, 1992). In humans, some studies have shown a cost of reproduction for mothers (Hruschka & Hagaman, 2015; Mace, 2000; Penn & Smith, 2007; Stieglitz et al., 2019; Tracer, 2002), although the most detailed, longitudinal study to date found little evidence for worsened nutritional or health status with increasing parity (Gurven et al., 2016). Such deviations from theoretical predictions may highlight other crucial socio-ecological inputs into human life history such as social support (see below), or point to ‘hidden’ costs such as reduced bone mineral density (Stieglitz et al., 2014) and fractures (Stieglitz et al., 2019). Thus, higher parity is expected to result in worse maternal condition (Prediction 1).

Quantity–quality tradeoffs were initially studied through the lens of clutch size in birds (Lack, 1947; Stearns, 1989), testing the prediction that increased offspring number in a given reproductive bout would decrease the survival of offspring from that bout. However, many experimental studies found that increased reproductive effort comes at a survival cost to parents rather than to offspring (Stearns, 2000; Vander Werf, 1992). In humans, with their predominantly singleton births, the relevant reproductive effort is the number of previous births and/or length of interbirth intervals. Indeed, offspring health and survival decrease with shorter birth intervals or increased number of siblings (Gibson & Lawson, 2011; Lawson et al., 2012; Mace, 2000; Penn & Smith, 2007). During demographic transitions, once extrinsic mortality rates drop and offspring survival depends more directly on parental investment; these tradeoffs are thought to play an important role in fertility reductions (Lawson et al., 2012; Lawson & Borgerhoff Mulder, 2016). Thus, higher parity is expected to result in worse offspring condition (Prediction 2).

The strength and fitness consequences of life-history tradeoffs vary among individuals as a function of their access to resources. Individuals with more resources are able to invest more in several life-history domains, thus resulting in phenotypic correlations among life-history traits and obscuring the underlying tradeoffs (van Noordwijk & de Jong, 1986). In humans, for instance, material wealth dampens the cost of reproduction (Hill & Hurtado, 1996; Hruschka & Hagaman, 2015; Sear et al., 2003) as wealthier people can afford to invest more in both longevity and fertility. A corollary of this observation is that individuals with greater access to resources, as proxied by social status, tend to have greater fitness (Alami et al., 2020; von Rueden & Jaeggi, 2016). Similarly, social support by husbands, kin or other social partners clearly plays a crucial role in human reproduction and can improve outcomes for mothers and offspring (Blurton Jones, 2016; Hill & Hurtado, 1996; Kramer, 2010; Mace & Sear, 2005; Sear & Mace, 2008). In the present study population, institutional support for women such as healthcare also improved over time, resulting in strong secular trends (Supplementary Material, Figure S1). Thus, life-history tradeoffs are often obscured by individual differences in resource access, with greater access resulting in weaker tradeoffs (Prediction 3) and greater overall fitness (Prediction 4).

Lastly, human reproduction is also subject to cultural norms that do not necessarily maximise reproductive success and may run counter to LHT predictions. Human reproduction has long been influenced by social institutions such as marriage, which is often arranged or at least influenced by parents and subject to conflicts of interest, as well as norms regarding fertility and ideal family size (Colleran, 2016; Walker, Hill, Flinn, & Ellsworth, 2011). Further, low-fertility norms, spreading through prestige- and conformity-biased learning are probably partly responsible for the recent worldwide declines in fertility (Richerson & Boyd, 2004), in addition to adaptive mechanisms such as quantity–quality tradeoffs or escalating investment in one's own embodied capital in ever more competitive mating markets (Colleran, 2016; Lawson & Borgerhoff Mulder, 2016; Shenk et al., 2016). Nevertheless, within a given subpopulation subject to common fertility tradeoffs and cultural norms, individuals with greater access to resources may still exhibit greater reproductive success (Alvergne & Lummaa, 2014; Mace, 2000).

Here we proposed to test these predictions using a newly transcribed dataset of *N* = 13,663 detailed birth records from the Basel maternity hospital (1896–1939), providing a snapshot of maternal and offspring conditions at the time of birth, the mother's age and parity, and several variables capturing her access to resources (see Table 1). In general, we expected more support for quantity–quality tradeoffs (Prediction 2) than for costs of reproduction for the mother (Prediction 1); this is partly because the prior literature is more decisive on the former, partly because our measurements of the latter are cruder (see ‘Data issues’). Further, we note a major caveat of the present data in that they contain no information on later-life outcomes, such as maternal and offspring longevity or embodied capital; it is therefore possible that no tradeoffs are detected because they occur later in life. Nevertheless, birth outcomes such as birth weight are generally predictive of later-life mortality and other outcomes (McCormick, 1985). This urban, industrialised population was in the mid to late stages of the demographic transition, with dramatic changes in mortality and fertility during the study period (see Figure S1). As such, it provides an important link between the types of populations more typically studied by evolutionary demographers, namely pre- and post-demographic transition societies (Blurton Jones, 2016; Colleran, 2016; Hill & Hurtado, 1996; Sear et al., 2016; Shenk et al., 2016).

**Table 1.** Overview of all variables

Variable name	Mean	SD	Range	Number missing
*General*				
Year	1918		1896–1939	0
Place of residence (Basel vs. others)	77.2% Basel			34
*Mother*				
Parity	2.17	1.82	1–19	26
Age at birth (years)	28.5	5.69	13–50	3
Age at menarche (years)	15.0	1.91	6–25	227
Mother height (cm)	157.2	6.26	101–196	2400
Nutritional status (1 = undernourished, 2 = normal, 3 = overweight)^a^	2.0	0.21	1–3	3006
Body shape (1 = gracile, 2 = normal, 3 = robust)^b^	2.3	0.81	1–3	395
Socio-economic position (1 = low, 2 = normal, 3 = high)	1.87	0.65	1–3	2913
Married (= has two last names on record)	87.2% yes			5
*Offspring*				
Gestational age (weeks)	39.7	2.44	13.9–44.9	418
Live birth	95.6% yes			33
Twins (yes/no)	99.8% no			489
Sex	52% male			48
Birthweight (g)	3245.1	547.08	90–5890	90
Body length (cm)	49.4	2.66	17–62	315
Head circumference (cm)	34.47	1.78	18.5–50	8917
Placenta weight (g)	631.03	137.86	150–1650	5070

a German: ‘*Ernährungszustand*’, literally nutritional status. A direct measure of current maternal condition.

b German: ‘*Knochenbau*’, literally bone build, from light to heavy. Refers more to skeletal robustness than to nutritional status. (Stieglitz et al., 2014, 2019).

## Methods

### Study population

In the nineteenth and twentieth centuries, Switzerland underwent large-scale social and economic changes in connection with industrialisation, urbanisation and the expansion of the service sector. After 1850 the city of Basel experienced rapid economic and population growth (see Appendix, Figure S1A) and developed from a typical trading town to a major chemical and pharmaceutical centre. As early as the end of the nineteenth century, Switzerland's gross domestic product put it among the wealthiest nations in Europe (Maddison, 2001). Between 1850 and 1950, the real wages of skilled workers in Basel increased by a factor of two (although temporarily interrupted by World War I, Figure S1B; Studer, 2008; Studer & Schuppli, 2008). Infant mortality rate (Figure S1C), which was generally higher in Swiss cities than in rural parts until the end of the nineteenth century, decreased at a steady rate for Basel, as did the stillbirth rate (Figure S1D) (Floris & Staub, 2019). Deaths owing to infectious diseases had been decreasing since the second half of the nineteenth century, also owing to large improvements in sanitation: access to clean water and flush sewage systems were provided in Basel from the 1860s and constantly expanded since then (Floris & Staub, 2019). Consequently, life expectancy increased (Floris, Höpflinger, Stohr, Studer, & Staub, 2019a; Floris, Staub, Stohr, & Woitek, 2019b). The average height of 19-year-old conscripts in Basel (covering 90–100% of male birth cohorts with Swiss citizenship) increased from 168.9 cm for young men born in 1894 to 173.8 cm for those born in 1938 (Figure S1E; Koepke et al., 2018; Schoch, Staub, & Pfister, 2012). Thus conventional measures of living standards, monetary as well as biological, describe a steady improvement in overall well-being during this study period.

The demographic transition was in progress in Basel from the last quarter of the nineteenth century, when first deaths and later also births per 1000 inhabitants started to decrease (Figure S1G). Mechanical and chemical contraception and birth control were increasingly used in Switzerland and Basel from 1900, with higher usage being suspected among women of more advantageous socioeconomic positions (Labhardt, 1930). An examination of the fertility of all Basel marriages concluded in 1921/1922 until the end of their reproductive phase in the 1940s showed that a total of 82% of those marriages had 0–2 children (25% of marriages remained childless; Labhardt, 1943). Basel thus had a comparatively low number of children per mother (2.00). Nevertheless, parities of up to 19 are present in the birth records (see Table 1). The average marriage age of the women was 25 years, and the first child was born on average in the third year after marriage (in 78% of the marriages within the first 6 years). Furthermore, the percentage of children breastfed increased from 90% in 1911 to a constant 95% after 1920 (Jann, 1934).

### Birth record data

The present data (see Table 1) come from the birth records of the university maternity hospital (‘Frauenspital’) of the canton Basel-Stadt. Detailed data on each childbirth have been routinely recorded since 1896. The register books are carefully maintained and incomplete records are very rare, although only a third of the records were archived owing to space constraints. Access to the protected individual data was allowed by the Staatsarchiv Basel-Stadt upon signed contractual agreement. After linking the sources, the data have been fully anonymised. The maternity hospital was founded in 1868 and by 1897 around 30% of all births in Basel took place there. This increased to 50% by 1912 and reached approximately 75% by 1930 (Figure S1F). Compared with other cities at that time the proportion of missed home births and births in other hospitals is therefore relatively low. Furthermore, birth records in Basel cover births from women of both high and low socioeconomic position, as well as complicated and problem-free childbirths. Nevertheless, each individual woman's birth history is likely to be incomplete owing to the missing record books.

The birth records contain information about birth outcomes as well as maternal condition (Table 1). Specifically, in addition to maternal age and marital status, height was routinely measured, age at menarche was recorded and the physician provided a crude assessment of the mother's nutritional status and body shape; both of the latter were coded as three-point scales reflecting increasing health and embodied capital (see ‘Data issues’).

Based on a continuous and unique entry number for each year, nearly 100% of the birth records could be linked to the birth register (inventory Sanität X8). From this second register, additional variables regarding the socioeconomic background of the father (if known) were available. Our measure of socioeconomic position (SEP) was derived from the occupations of the father or the mother using the classification in Schüren (1989). The results of this classification are six categories, which we aggregated to three groups: low (1, 2), medium (3, 4) and high SEP (5, 6). We use the SEP of the father, if available, and otherwise the SEP of the mother. However, records where mothers indicated ‘housewife’ and the SEP of the father was not available were treated as missing SEP data. Comparing the shares of father's occupation with the census shares in 1920 for male occupation groups shows that our sample closely reflects the SEP distribution in this population (Supplementary Table S1).

### Data analysis

We used a Bayesian multivariate, multilevel model implemented in the ‘*brms*’ package (Bürkner, 2017) in R. A multivariate model allowed us to simultaneously analyse multiple related outcomes (e.g. birth weight, birth length, etc.) in a single model while accounting for any residual correlations among them not captured by our primary predictors; for instance, all else being equal, heavier babies are also expected to be longer, have larger heads, etc. Accounting for such general correlations among outcome variables thus made our analysis more accurate and conservative, because parameter estimates were pooled across outcomes and extreme estimates shrunk towards the mean (McElreath, 2020), compared with treating all outcomes as independent in separate models. Fitting these models in *brms* also facilitated appropriate modelling of nonlinear effects using splines for variables with probably fluctuating effects (year) and monotonic effects (Bürkner & Charpentier, 2020) for ordinal variables (SEP), as well as imputing missing data and propagating the associated uncertainty into final parameter estimates (McElreath, 2020; Zhou & Reiter, 2010); specifically, we imputed 10 complete datasets and ran our models on each of them and then combined the posterior samples (Zhou & Reiter, 2010). Based on our experience transcribing the data and inspecting missingness patterns, we have no reason to believe that missingness was non-random, and Figure S2 shows that imputed values have high plausibility as evidenced by density plots overlapping with the observed data (van Buuren & Groothuis-Oudshoorn, 2011). Imputation makes no additional assumptions on patterns of missingness compared with complete-case analysis, but has the huge advantage of using all of the valuable information rather than discarding incomplete cases, thus generally leading to less biased estimates (McElreath, 2020). Further, Bayesian estimation allowed us to use regularising priors to impose conservatism on parameter estimates and reduce our risk of inferential errors (McElreath, 2020).

Our approach also accounted for unobserved variation at various levels that may affect both parity and maternal/offspring condition and is likely to mask tradeoffs. As noted in the introduction, unobserved variation in energetic condition or access to resources commonly masks life-history tradeoffs (van Noordwijk & de Jong, 1986), including in humans (Sear, 2020; Sear et al., 2003). Technically this problem is referred to as endogeneity bias, which arises from the residuals of a model being correlated with a predictor (Steele, Vignoles, & Jenkins, 2007); here, condition correlates with parity owing to unobserved variation in factors affecting both. This bias can be resolved by jointly estimating the processes that determine parity and maternal/offspring condition and letting the residuals from their respective linear predictors be correlated in a multivariate model (Steele et al., 2007). If these correlations are different from 0 this indicates that endogeneity was indeed present, and the sign of the correlation may indicate underlying variation in energetic condition (if parity and outcomes are positively correlated) or provide further evidence of tradeoffs not accounted for by the parity fixed effect (for negative correlations). The same logic applies to random intercepts for mothers, which were also allowed to be correlated across all outcomes in our model. Specifically, the mother random effect captures consistent individual differences in birth outcomes among mothers, for women appearing more than once in the data, independent of our fixed effects. Such individual differences are expected to result from unobserved factors such as overall phenotypic quality (often referred to as ‘frailty’ by demographers) that would otherwise mask underlying tradeoffs within mothers’ reproductive careers (Doblhammer & Oeppen, 2003). More specifically, akin to the correlated residuals mentioned above, a positive correlation of mother ID intercepts across outcomes, e.g. reflecting higher than average offspring condition and (age-specific) parity, would provide evidence of individual variation in frailty, while a negative correlation would provide further evidence for tradeoffs that is not accounted for by the parity fixed effect. Place of residence (Basel, *n* = 10,515; others, *n* = 3114) was included as a fixed effect to capture any unobserved variation in living conditions or social norms not accounted for by SEP and year. In sum, endogeneity bias stemming from phenotypic correlation can be resolved by using multivariate, multilevel models with correlated residuals and random effects, thus increasing the chance of detecting tradeoffs.

The general formula for our multivariate model is given below. For simplicity, we illustrate here only one measure of maternal and offspring condition, but the full set of measures included nutritional status and body shape for maternal condition, and live birth, birth weight, body length, gestational age, head circumference and placental weight for offspring condition (see Table 1). All of these outcome variables were modelled using Gaussian distributions except for nutritional status and body shape (both cumulative ordinal distributions), the probability of a live birth (Bernoulli) and parity (Poisson). Gaussian variables were standardised prior to analysis to facilitate comparison and interpretation of model parameters.

Our model structure for the ordinal response of nutritional status for birth record *i* is given by
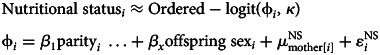


Here ϕ is the linear predictor for the probability of observing a mother across the *κ* categories, given the global intercepts *μ*_*k*_ = {*μ*_bad_, *μ*_normal_, *μ*_good_} for each nutritional status. The log-cumulative-odds for observing a mother in any particular category *k* are therefore given by *μ*_*k*_ − ϕ. The *β* are regression coefficients for each of *x* fixed effects, which include the main predictor parity as well as other covariates including maternal age, place of residence, year, socio-economic position (SEP), marital status, maternal height, age of menarche, twin birth and offspring sex (see Tables 1 and 2). Here, height and age of menarche reflect nutrition and general conditions during a woman's development (taller and earlier menarche = better conditions; Bogin, 1999; Stearns & Koella, 1986), while SEP, marital status and year reflect current conditions (higher SEP and married, later years = better); twin and male births (Galbarczyk, Klimek, Nenko, & Jasienska, 2019) are expected to impose additional costs on mothers. Note that SEP was modelled as a monotonic effect (Bürkner & Charpentier, 2020) and year using a spline to allow for nonlinearity. *A priori*, we expected year to be nonlinear because, while conditions generally improved over the study period, World War I (1914–1918) and the Spanish flu (1918) presented major shocks that could have disrupted a linear increase. Random intercepts for nutritional status *μ*^NS^ are specified for the mother, along with an observation-level random effect *ɛ* capturing residual variation (also known as overdispersion; Nakagawa, Johnson, & Schielzeth, 2017), which can then be correlated across outcomes to address the endogeneity bias (Steele et al., 2007) as specified below.

**Table 2.** List of predictions and relevant model parameters that test them. The column on the right indicates qualitative support for each prediction. Note that for Predictions 1 and 2, the main effects of parity (*β*_1_) are the most pertinent tests; negative correlations (*ρ*) between residuals or mother-level intercepts of parity and outcomes would indicate energetic tradeoffs, but caused by some unobserved variable rather than by parity itself.

Prediction	Relevant parameters in the model	Supported?
1: Tradeoff fertility maternal health (‘cost of reproduction’)	*β*_1_: Negative association of parity with nutritional status and body shape *ρ*: Negative correlations ( 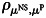 ) between residuals of parity ( 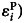 and nutritional status ( 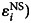 and for mother-level random intercepts for parity ( 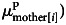 and nutritional status ( 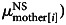 ; the same for body shape	Unable to test
2: Tradeoff fertility offspring health (‘quantity–quality tradeoff’)	*β*_1_: Negative association of parity with birth weight, birth length, live birth, gestational age, placenta weight, and head circumference *ρ*: Negative correlation ( 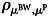 ) between residuals of parity ( 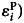 and birth weight ( 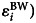 and for mother-level random intercepts for parity ( 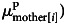 and birth weight ( 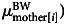 ; the same for other offspring outcomes	No (Fig. 1)
3: Reduced tradeoff with greater access to resources	Other *β*s: interactions between parity and SEP, year, height, age of menarche, marital status in predicting mother and offspring outcomes, such that negative effect of parity is weaker for women with better current conditions (higher SEP, married, later years) or conditions experienced during their development (taller women, women with earlier menarche)	No (Fig. 2)
4: Higher overall fitness with greater access to resources	Other *β*s: higher SEP, being married, taller height and earer age of menarche associated with higher parity, controlling for year (and thereby prevalent social norms)	Mixed (Fig. 3)
Further expected associations	Other *β*s: negative association between twin birth, male sex and mother and offspring outcomes	Unable to test/yes (Fig. 4)

Similarly, our basic model structure for birth weight is given by
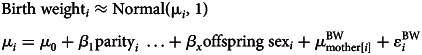


Here the global intercept is represented by *μ*_0_. We fix the scale of the residual standard deviation (*σ* = 1) because this parameter is redundant with the observation-level random effect *ɛ*. While mathematically equivalent, this parameterisation facilitates correlating residual error across the Gaussian and non-Gaussian responses.

Finally, for our count measure of parity the basic model structure is
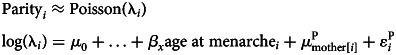


This model included the same fixed effects except twin birth and offspring sex as these are not relevant predictors of parity. To account for associations between the response measures, we correlate maternal and observation-level random effects. For example, we estimate correlations between the maternal random effects such that:
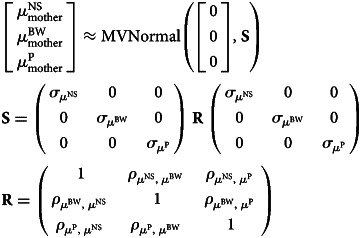


The covariance matrix **S** is parameterised such that inference is made directly on the correlation matrix **R** using the standard deviations (*σ*) of random effects (McElreath, 2020). The same parameterisation is used for the correlations of observation-level random effects. These correlation matrices can also provide evidence for hypothesised energetic tradeoffs between traits, e.g. a negative correlation between residuals of parity and birth weight (see Table 2), although these tradeoffs would be caused by an unobserved variable rather than by parity *per se*.

Common priors were placed on all model parameters to conservatively regularise our estimates and thus enhance the robustness of our inferences (Gelman et al., 2013; McElreath, 2020). In particular, we specified
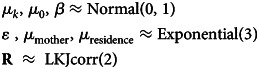


Table 2 lists all predictions and specifies which model parameters constitute direct tests of these predictions. We further provide expected associations between control variables and outcomes that do not constitute direct tests of our predictions.

To assess support for predictions, we use fully Bayesian, quantitative inference (Gelman et al., 2013; McElreath, 2020; McShane, Gal, Gelman, Robert, & Tackett, 2019). For each estimated parameter that tests a prediction, we plot the posterior distribution and report the proportion of the posterior that supports the prediction (e.g. denoted as *P*_<0_ for predicted negative associations), thus directly quantifying the degree of support for the prediction. Note that in contrast to frequentist *p*-values, which indicate the probability of observing the data under the null hypothesis, *p*(data|H_0_), the reported posterior probabilities estimate the support of hypothesised positive or negative associations given the data, i.e. *p*(H_1_|data). In short, larger values of *P*_<0_ etc. indicate greater support for our predictions, which allows readers to quantitatively evaluate the results rather than relying on dichotomous inference.

### Data issues

#### Representativeness of the sample

Despite a good representation of all births and socioeconomic positions, there are likely to be some biases in this dataset. Specifically, the sample is probably biased towards women of lower SEP in the earlier years when the percentage of hospital births was relatively low, before about 1910 when the SEP distribution of the hospital births begins to match that of the general census (conducted in 1910, 1920, 1930 and 1940). Ideally these biases would be dealt with by first conducting our analyses on the full dataset to retain maximum statistical power, but then randomly selecting birth records from each decade to match the SEP distribution of the closest census. However, in reality computational constraints prevented us from doing this.

#### Matching mothers

Some women appear several times in the dataset but no personal identifier was available to match records. Furthermore, date of birth was incompletely recorded (4950 records missing). Hence we matched records of the same women in this dataset by name and birth year (only three missing). To account for slight variations in spelling, we calculated string distances between the first name and maiden name (which was consistently entered even after marriage) of a focus record and all other records in the dataset, and only considered records where both first and maiden name differed by maximally one letter as possible matches. We further required possible matches to have the same birth year. Lastly, we imposed relevant logical criteria on matched cases, namely the mother's parity had to be lower if the year of giving birth was earlier but higher if the year was later. This stringent approach resulted in 13,189 unique mother IDs; in other words, only 448 mother IDs appeared more than once. However, relaxing the criteria, e.g. to allow names to differ by up to two letters, resulted in numerous false matches, and still only 13,089 unique mother IDs. Hence we stuck to the stringent matching criteria.

#### Coding mother outcomes

Unlike offspring outcomes, which were measured quantitatively (e.g. birth weight in grams), mother outcomes were somewhat qualitatively assessed, resulting in a large number of labels. We condensed these to three-point scales to reflect continuous measures of nutritional status (ranging from undernourished to overnourished) and body shape (ranging from gracile to robust), respectively. Arguably, this coding could have been done at a finer scale; for instance, the ‘gracile’ level of body shape includes one potential case of dwarfism (‘Zwergenwuchs’) and several dozen cases of rickets (‘rachitisch’) as well as more general gracile or slender body types. However, we prioritised an unambiguous increase from levels 1 to 3 and good representation in each level to maximise our chances of detecting general trends in these variables.

## Results

### Relation to Stage 1 manuscript and proposed model

The accepted Stage 1 manuscript, the anonymised dataset and all R code for reproducing the results can be accessed on the Open Science Framework: https://osf.io/v2s43/files/. We had to make a few changes to the model proposed in Stage 1 in order to achieve convergence. Most importantly, we had to exclude mother outcomes (i.e. nutritional status and body shape) because there was simply not enough variation in these variables (see also Table 1), hence we were unfortunately unable to test Prediction 1. Furthermore, residual and random-effect correlations between parity and outcomes at the within- and between-mother levels could only be estimated after reducing the dataset to mothers who were observed more than once (*n* = 953 birth records). Hence we prioritised the presentation of results from this reduced model (henceforth Model 1) and we fit a separate model on mothers observed only once (henceforth Model 2) to check the robustness of our inferences. Minor changes relative to the originally proposed model included changing location from a random effect to a fixed effect since it only contained two levels (Basel vs. other), and using exponential rather than half-Cauchy priors for variance components. Model 2 had a much larger sample size (*n* = 12,710 birth records), but lacked a mother random effect and residual correlations between parity and outcomes (consequently, σ was estimated rather than fixed); furthermore, age fixed effects for all outcomes except parity and live births had to be removed from Model 2 to achieve convergence. The results from Model 2 are presented in Figures S2–S5, and agree with Model 1 unless otherwise noted. Owing to computational constraints (each model took days to run), we limited the number of imputed datasets to 10 and refrained from fitting random slopes and doing any formal model comparisons among nested model sets; instead we ran a few additional robustness checks as described below.

### Prediction 1

This prediction could unfortunately not be tested because there was insufficient variation in the maternal condition variables and they had to be removed from the model.

### Prediction 2

**Figure 1.**
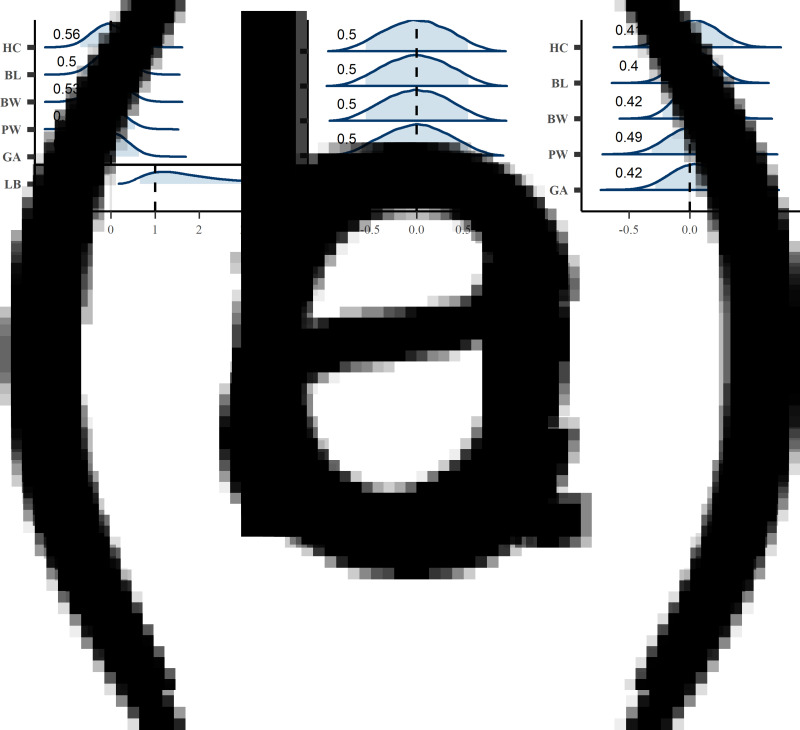
No effect of parity on offspring outcomes. (a) The main effect of parity, (b) the residual correlation between parity and offspring outcomes within mothers and (c) the residual correlation between mothers. Plotted are the posterior distributions with 90% credible interval, and numbers give the proportion of the posterior that supports the prediction (e.g. the proportion of the posterior <0, or *P*_<0_). LB, Probability of live birth (expressed as an odds ratio); GA, gestational age; PW, placenta weight; BW, birth weight; BL, birth length; HC, head circumference.

Overall, there was no support for prediction 2. Specifically, parity did not have a negative effect on any of the offspring outcomes (Figure 1a), and there were no negative residual correlations between parity and offspring outcomes, either within (Figure 1b) or between mothers (Figure 1c). Thus, there was no evidence for quantity–quality tradeoffs (main effect of parity) or other energetic tradeoffs caused by unobserved variables (residual correlations).

### Prediction 3

**Figure 2.**
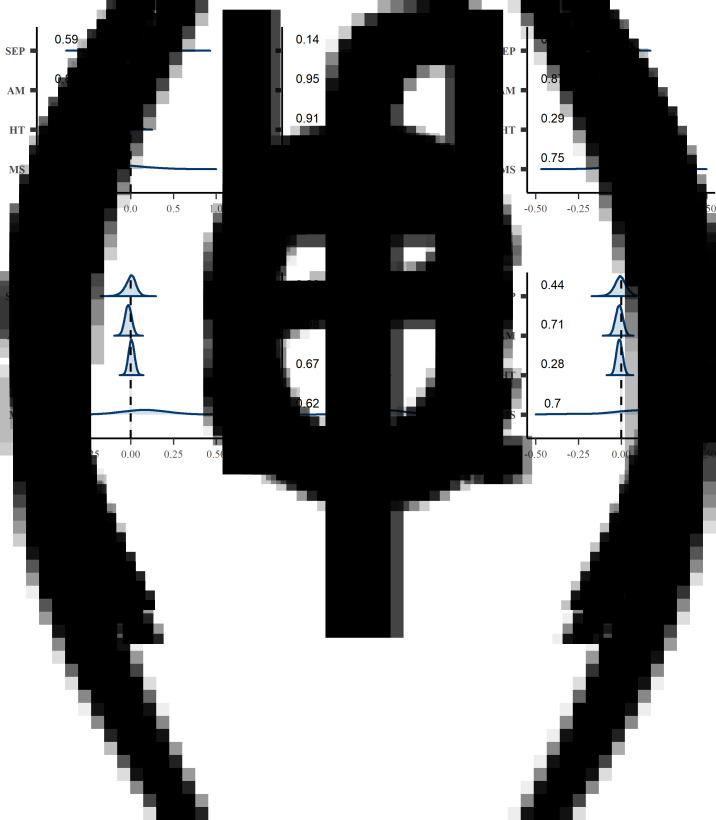
Interactions between parity and maternal condition on offspring outcomes, namely (a) probability of live birth, (b) gestational age, (c) placenta weight, (d) birth weight, (e) birth length and (f) head circumference. Maternal condition was proxied by marital status (MS, indicating being married vs. not married), height (HT), age of menarche (AM), socio-economic position (SEP) and year. Plotted are the posterior distributions with 90% credible interval, and numbers give the proportion of the posterior that supports the prediction (e.g. the proportion of the posterior <0, or *P*_<0_). Estimates for the interaction between parity and the year spline included three parameters, which are not shown here owing to a lack of clear interpretability.

Prediction 3, i.e. an alleviation of quantity–quality tradeoffs in mothers with better access to resources, was also not supported as the posteriors for most of the relevant interaction terms indicated very small effect sizes that largely overlapped with 0 (Figure 2). The exception was age of menarche, wherein women with a later age of menarche, indicating worse childhood nutritional conditions, experienced more negative effects of parity on most offspring outcomes. However, these effects were not large: for instance, all else being equal, birth length (the best supported interaction, with *P*_<0_ = 0.98) was predicted to change by 0 standard deviations (95% CI = −0.73–0.76) with each additional parity for women with average age of menarche, and by −0.04 SDs (95% CI = −0.77–0.72) for women with +1 SD higher age of menarche. Furthermore, the age of menarche interaction was not well supported in Model 2 (Figure S4).

### Prediction 4

**Figure 3.**
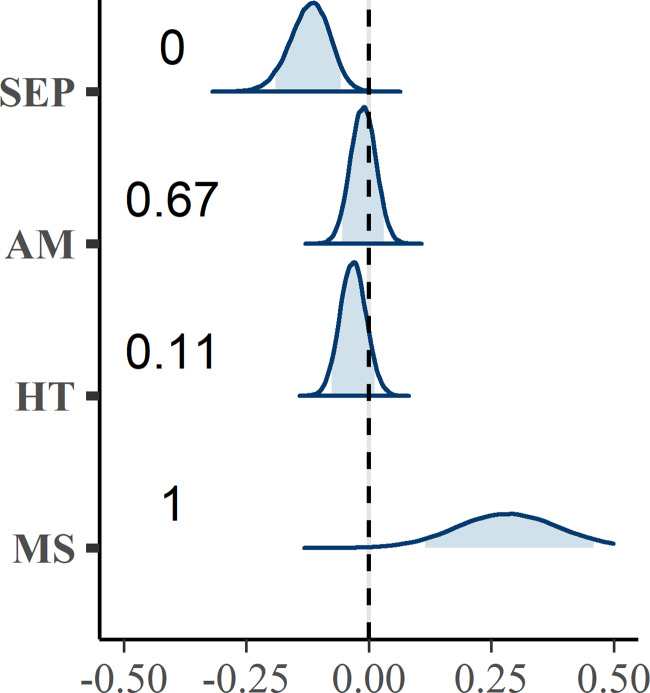
Effects of maternal condition on parity. Maternal condition was proxied by marital status (MS), height (HT), age of menarche (AM), and socio-economic position (SEP). Plotted are the posterior distributions with 90% credible interval, and numbers give the proportion of the posterior that supports the prediction (e.g. the proportion of the posterior <0, or *P*_<0_)

We predicted that women should translate better access to resources into higher parity, all else being equal. However, only marital status (i.e. being married rather than unmarried) was associated with higher parity, while higher SEP was strongly associated with *lower* parity; the relationship with the mother's height was also negative, while that with age of menarche went in the right direction – earlier age of menarche = higher parity – but was highly uncertain (Figure 3). In Model 2 all of these associations went in the same direction with no posteriors overlapping with 0, but the effect sizes for height and age of menarche were extremely small (Figure S5).

### Further predicted associations

**Figure 4.**
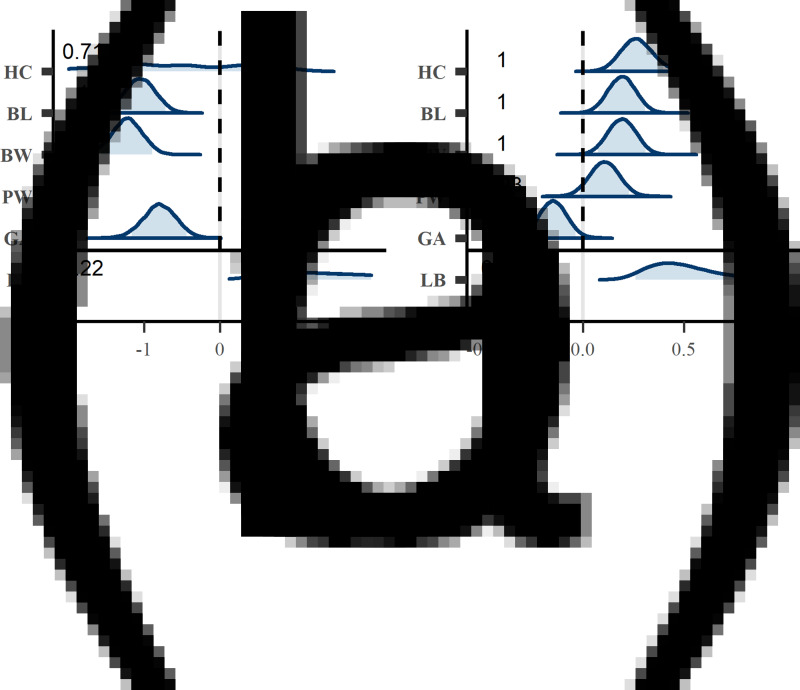
Effects of (a) twin births (twins vs. non-twins) and (b) offspring sex (male vs. female) on offspring condition. Plotted are the posterior distributions with 90% credible interval and numbers give the proportion of the posterior that supports the prediction (e.g. the proportion of the posterior <0, or *P*_<0_). LB, Probability of live birth (expressed as an odds ratio); GA, gestational age; PW, placenta weight; BW, birth weight; BL, birth length; HC, head circumference.

We predicted negative effects of twinning and male sex on mother and to some extent offspring outcomes owing to increased energetic demands on mothers. There were no twin births to mothers observed more than once, hence we present the results for this prediction from Model 2, in which twin births were associated with shorter gestation, much larger placentas, and lower birth weight and length (Figure 4a). Male offspring were larger in every dimension, were born after shorter pregnancies, and were more likely to be stillborn (Figure 4b), all of which indicates greater energetic demands on mothers.

### Robustness checks

To ensure the robustness of our inferences, we ran several alternative versions of Model 1. We excluded all interaction terms and/or all main effects except parity to reduce collinearity and confounding, let parity have non-linear effects, accounted for heteroscedasticity by letting sigma vary as a function of parity, excluded unmarried women and first births because they generally have worse offspring outcomes, and/or excluded preterm births and unrealistic ages of menarche (>2 SD below the mean) as well as stillbirths and twins. These models did not meaningfully change our results (details not reported).

## Discussion

Tradeoffs between fertility and survival have played an important role in adaptive explanations of demographic transitions, as women may reduce their fertility when having more children is costly for themselves or their offspring (Gibson & Lawson, 2011; Lawson et al., 2012; Lawson & Borgerhoff Mulder, 2016; Mace, 2000; Penn & Smith, 2007). Here we tested for such tradeoffs using historical birth records from a period of rapid demographic transition (see Figure S1). Despite our best efforts to account for common statistical issues (Doblhammer & Oeppen, 2003; Steele et al., 2007; van Noordwijk & de Jong, 1986), we found no evidence for tradeoffs between offspring quantity and quality; others have argued that this tradeoff does not actually account for much variation in human fertility (Lawson & Borgerhoff Mulder, 2016). We were unable to test for negative effects of parity on maternal condition, but note that some of the best studies have found no evidence for this (Gurven et al., 2016). In addition, instead of converting greater access to resources into higher fertility (Alvergne & Lummaa, 2014; Mace, 2000), women of higher socio-economic position had fewer children than others living in the same place at the same time.

There are several limitations to this study. First, crucial variables were relatively crude, including maternal condition (preventing us from testing Prediction 1), SEP (and thereby resource access) and location (as life-history strategies may cluster by neighborhood within a city: Daly & Wilson, 1997). Second, the data provide only a snapshot of offspring condition at birth, and even though birth outcomes are predictive of later-life health (McCormick, 1985), our analysis may miss some later-life costs, especially if they are economic rather than biological (e.g. the cost of schooling). Furthermore, quantity–quality tradeoffs may perhaps be weaker at lower parities, as observed here, although others have argued the opposite (Lawson & Borgerhoff Mulder, 2016). Ultimately, the lack of quantity–quality tradeoffs reflects a time of rapid economic growth and reductions in extrinsic mortality (e.g. through improvements in sanitation and public health) that may have lifted energetic constraints on this population.

If not quantity–quality tradeoffs, what else may explain fertility reductions in Basel? First, there could be costs of reproduction to mothers, if not in terms of health or longevity (Gurven et al., 2016) then perhaps in terms of investment in their own embodied capital in an increasingly competitive labour and marriage market (Shenk et al., 2016). The latter hypothesis could in theory be tested by using mothers’ SEP to predict parity (in addition to fathers’), although most observations only state ‘housewife’, which probably obscures relevant variation in embodied capital. Second, fertility reductions could be caused by maladaptive social norms initially adopted by people of higher SEP before spreading by prestige- and eventually conformity-biased transmission (Colleran, 2016; Richerson & Boyd, 2004). To further distinguish between these hypotheses, we tested whether the association between SEP and parity changes over time; the embodied capital hypothesis would predict it to become more negative as competition intensifies, while the social norms hypothesis would predict it to weaken as low-fertility norms spread from high SEPs to the general population. However, adding an interaction between SEP and year to Model 1 and inspecting raw data failed to provide evidence for changes in the SEP–parity association over time (details not reported), leaving the opening question of this paragraph with several possible answers.

In sum, our study failed to find evidence for quality–quantity tradeoffs in a population undergoing rapid fertility decline, suggesting that life-history tradeoffs did not play a major role at this stage of the demographic transition in historical Basel.

## Supplementary material

For supplementary material accompanying this paper visit https://doi.org/10.1017/ehs.2022.2. click here to view supplementary material

## Research transparency and reproducibility

The accepted Stage 1 manuscript, the anonymised dataset and all R code for reproducing the results can be accessed on the Open Science Framework: https://osf.io/v2s43/files/.

## References

[ref1] Alami, S., Rueden, C. Von, Seabright, E., Kraft, T. S., Blackwell, A. D., Stieglitz, J., … Gurven, M. D. (2020). Mother's social status is associated with child health in a horticulturalist population. Proceedings of the Royal Society B – Biological Sciences, 287, 20192783. 10.1098/rspb.2019.2783 PMC7126073 32156217

[ref2] Alvergne, A., & Lummaa, V. (2014). Ecological variation in wealth−fertility relationships in Mongolia: The ‘central theoretical problem of sociobiology’ not a problem after all? Proceedings of the Royal Society B: Biological Sciences, 281(1796), 20141733. 10.1098/rspb.2014.1733 PMC4213645 25320175

[ref3] Blurton Jones, N. G. (2016). Demography and evolutionary ecology of the Hadza hunter–gatherers. Cambridge University Press.

[ref4] Bogin, B. (1999). Patterns of human growth (2nd ed.). Cambridge University Press.

[ref5] Bürkner, P.-C. (2017). brms: An R package for Bayesian multilevel models using Stan. Journal of Statistical Software, 80. doi: 10.18637/jss.v080.i01.

[ref6] Bürkner, P. C., & Charpentier, E. (2020). Monotonic effects: A principled approach for including ordinal predictors in regression models. British Journal of Mathematical and Statistical Psychology. doi: 10.1111/bmsp.12195 31943157

[ref7] Charnov, E. L. (1991). Evolution of life history variation among female mammals. Proceedings of the National Academy of Sciences of the United States of America, 88(4), 1134–1137. 10.1073/pnas.88.4.1134 1996315 PMC50971

[ref8] Charnov, E. L., & Berrigan, D. (2005). Why do female primates have such long lifespans and so few babies? Or Life in the slow lane. Evolutionary Anthropology: Issues, News, and Reviews, 1(6), 191–194. 10.1002/evan.1360010604

[ref9] Colleran, H. (2016). The cultural evolution of fertility decline. Philosophical Transactions of the Royal Society B: Biological Sciences, 371(1692), 20150152. 10.1098/rstb.2015.0152 PMC4822432 27022079

[ref10] Daly, M., & Wilson, M. (1997). Life expectancy, economic inequality, homicide, and reproductive timing in Chicago neighborhoods. British Medical Journal, 314, 1271–2174. 9154035 10.1136/bmj.314.7089.1271 PMC2126620

[ref11] Doblhammer, G., & Oeppen, J. (2003). Reproduction and longevity among the British peerage: The effect of frailty and health selection. Proceedings of the Royal Society B: Biological Sciences, 270(1524), 1541–1547. 10.1098/rspb.2003.2400 PMC1691410 12908973

[ref12] Floris, J., Höpflinger, F., Stohr, C., Studer, R., & Staub, K. (2019a). Wealthier – older – taller: Measuring the standard of living in Switzerland since the 19th century. Schweizerische Zeitschrift Für Geschichte, 69(2), 207–232.

[ref13] Floris, J., & Staub, K. (2019). Water, sanitation and mortality in Swiss towns in the context of urban renewal in the late nineteenth century. The History of the Family, 24(2), 249–276. 10.1080/1081602X.2019.1598460

[ref14] Floris, J., Staub, K., Stohr, C., & Woitek, U. (2019b). Changes in mortality in Switzerland, 1880–1910. In F. Z. Juan, G. Hürlimann, L. Lorenzetti, & H.-U. Schiedt (Eds.), Schweizerisches Jahrbuch für Wirtschafts- und Sozialgeschichte: Texte und Zahlen (pp. 69–91). Chronos.

[ref15] Galbarczyk, A., Klimek, M., Nenko, I., & Jasienska, G. (2019). Sons may be bad for maternal health at older age: New evidence for costs of reproduction in humans. The Journals of Gerontology: Series A, 74(5), 648–651. 10.1093/gerona/gly190 30137222

[ref16] Gelman, A., Carlin, J. B., Stern, H. S., Dunson, D. B., Vehtari, A., & Rubin, D. B. (2013). Bayesian data analysis *(3rd edn)*. Chapman and Hall.

[ref17] Gibson, M. A., & Lawson, D. W. (2011). ‘Modernization’ increases parental investment and sibling resource competition: Evidence from a rural development initiative in Ethiopia. Evolution and Human Behavior, 32(2), 97–105. 10.1016/j.evolhumbehav.2010.10.002

[ref18] Gurven, M., Costa, M., Ben Trumble, B., Stieglitz, J., Beheim, B., Eid Rodriguez, D., … Kaplan, H. (2016). Health costs of reproduction are minimal despite high fertility, mortality and subsistence lifestyle. Scientific Reports, 6(1), 30056. 10.1038/srep30056 27436412 PMC4951795

[ref19] Healy, K., Ezard, T. H. G., Jones, O. R., Salguero-Gómez, R., & Buckley, Y. M. (2019). Animal life history is shaped by the pace of life and the distribution of age-specific mortality and reproduction. Nature Ecology and Evolution, 3(August). 10.1038/s41559-019-0938-7 31285573

[ref20] Hill, K., & Hurtado, A. M. (1996). Ache life history: The ecology and demography of a foraging people. Aldine de Gruyter.

[ref21] Hruschka, D. J., & Hagaman, A. (2015). The physiological cost of reproduction for rich and poor across 65 countries. American Journal of Human Biology, 659(November 2014), 654–659. 10.1002/ajhb.22707 25809493

[ref22] Jann, M. (1934). Die Geburten der letzten 20 Jahre in Basel-Stadt. Eine statistische Studie aus den Geburtstabellen der Stadt Basel 1911–1930. Buchdruckerei Huber.

[ref23] Knowles, S. C. L., Nakagawa, S., & Sheldon, B. C. (2009). Elevated reproductive effort increases blood parasitaemia and decreases immune function in birds: A meta-regression approach. Functional Ecology, 23(2), 405–415. 10.1111/j.1365-2435.2008.01507.x

[ref24] Koepke, N., Floris, J., Pfister, C., Rühli, F. J., & Staub, K. (2018). Ladies first: Female and male adult height in Switzerland, 1770–1930. Economics & Human Biology, 29, 76–87. 10.1016/j.ehb.2018.02.002 29486413

[ref25] Kramer, K. L. (2010). Cooperative breeding and its significance to the demographic success of humans. Annual Review of Anthropology, 39(1), 417–436. 10.1146/annurev.anthro.012809.105054

[ref26] Labhardt, A. (1930). Geburtenrückgang, Geburtenregelung. Benno Schwabe.

[ref27] Labhardt, A. (1943). Fertilität und Sterilität Basler Ehen. Schweizerische Medizinische Wochenschrift, 7, 91–97.

[ref28] Lack, D. (1947). The significance of clutch size. Ibis, 89, 302–352.

[ref29] Lawson, D. W., Alvergne, A., & Gibson, M. A. (2012). The life-history trade-off between fertility and child survival. Proceedings of the Royal Society B: Biological Sciences, 279(October), 4755–4764. 10.1098/rspb.2012.1635 PMC3497086 23034700

[ref30] Lawson, D. W., & Borgerhoff Mulder, M. (2016). The offspring quantity–quality trade-off and human fertility variation. Philosophical Transactions of the Royal Society B: Biological Sciences, 371(1692), 20150145. 10.1098/rstb.2015.0145 PMC4822425 27022072

[ref31] Mace, R. (2000). Evolutionary ecology of human life history. Animal Behaviour, 59(1), 1–10. 10.1006/ANBE.1999.1287 10640361

[ref32] Mace, R., & Sear, R. (2005). Are humans cooperative breeders? In E. Voland, A. Chasiotis, & W. Schiefenhövel (Eds.), Grandmotherhood: The evolutionary significanc of the second half of female life (pp. 143–159). The State University.

[ref33] Maddison, A. (2001). The world economy. A millenial perspective. OECD.

[ref34] McCormick, M. C. (1985). The contribution of low birth weight to infant mortality and childhood morbidity. New England Journal of Medicine, 312, 82–90. 3880598 10.1056/NEJM198501103120204

[ref35] McElreath, R. (2020). Statistical rethinking: A Bayesian course with examples in R and Stan *(2nd edn)*. CRC Press.

[ref36] McShane, B. B., Gal, D., Gelman, A., Robert, C., & Tackett, J. L. (2019). Abandon statistical significance. American Statistician, 73(suppl. 1), 235–245. 10.1080/00031305.2018.1527253

[ref37] Nakagawa, S., Johnson, P. C. D., & Schielzeth, H. (2017). Coefficient of determination R2 and intra-class correlation coefficient from generalized linear mixed-effects models revisited and expanded. Journal of the Royal Society Interface, 14, 20170213. 10.1101/095851 28904005 PMC5636267

[ref38] Nettle, D., Gibson, M. A., Lawson, D. W., & Sear, R. (2013). Human behavioral ecology: Current research and future prospects. Behavioral Ecology, 24(5), 1031–1040. 10.1093/beheco/ars222

[ref39] Penn, D. J., & Smith, K. R. (2007). Differential fitness costs of reproduction between the sexes. Proceedings of the National Academy of Sciences, 104(2), 553–558. 10.1073/PNAS.0609301103 PMC1766423 17192400

[ref40] Richerson, P. J., & Boyd, R. (2004). Not by genes alone: How culture transformed human evolution. University of Chicago Press. http://books.google.com/books?hl=en&lr=&id=dU-KtEVgK6sC&pgis=1

[ref41] Roff, D. A. (1993). Evolution of life histories: Theory and analysis. Chapman & Hall.

[ref42] Santos, E. S. A., & Nakagawa, S. (2012). The costs of parental care: A meta-analysis of the trade-off between parental effort and survival in birds. Journal of Evolutionary Biology, 25(9), 1911–1917. 10.1111/j.1420-9101.2012.02569.x 22830387

[ref43] Schoch, T., Staub, K., & Pfister, C. (2012). Social inequality and the biological standard of living: An anthropometric analysis of Swiss conscription data, 1875–1950. Economics and Human Biology, 10(2), 154–173. 10.1016/j.ehb.2011.05.001 21641285

[ref44] Schüren, R. (1989). Soziale Mobilität. Muster, Veränderungen und Bedingungen im 19. Und 20. Jahrhundert. Scripta Mercaturae Verlag.

[ref45] Sear, R. (2020). Do human ‘life history strategies’ exist? Evolution and Human Behavior, 41(6), 513–526.

[ref46] Sear, R., Lawson, D. W., Kaplan, H., & Shenk, M. K. (2016). Understanding variation in human fertility: what can we learn from evolutionary demography? Philosophical Transactions of the Royal Society B: Biological Sciences, 371(1692), 20150144. 10.1098/rstb.2015.0144 PMC4822424 27022071

[ref47] Sear, R., & Mace, R. (2008). Who keeps children alive? A review of the effects of kin on child survival. Evolution and Human Behavior, 29(1), 1–18. http://www.sciencedirect.com/science/article/B6T6H-4RB0P7G-1/2/77fcab7321c654cd846e136351afefd2

[ref48] Sear, R., Mace, R., & McGregor, I. A. (2003). A life history approach to fertility rates in rural Gambia: Evidence for trade-offs or phenotypic correlations? In The Biodemography of human reproduction and fertility (pp. 135–160). Springer US. 10.1007/978-1-4615-1137-3_7

[ref49] Shenk, M. K., Kaplan, H. S., & Hooper, P. L. (2016). Status competition, inequality, and fertility: Implications for the demographic transition. Philosophical Transactions of the Royal Society, B, 371(1692), 20150150. 10.1098/rstb.2015.0150 PMC4822430 27022077

[ref50] Stearns, S. C. (1989). Trade-offs in life-history evolution. Functional Ecology, 3(3), 259–268.

[ref51] Stearns, S. C. (1992). The evolution of life histories. Oxford University Press.

[ref52] Stearns, S. C. (2000). Life history evolution: successes, limitations, and prospects. Naturwissenschaften, 87(11), 476–486. 10.1007/s001140050763 11151666

[ref53] Stearns, S. C., & Koella, J. C. (1986). The evolution of phenotypic plasticity in life-history traits: Predictions of reaction norms for age and size at maturity. Evolution, 40(5), 893–913. 10.1111/j.1558-5646.1986.tb00560.x 28556219

[ref54] Steele, F., Vignoles, A., & Jenkins, A. (2007). The effect of school resources on pupil attainment: A multilevel simultaneous equation modelling approach. Journal of the Royal Statistical Society. Series A: Statistics in Society, 170(3), 801–824. 10.1111/j.1467-985X.2007.00476.x

[ref55] Stieglitz, J., Beheim, B. A., Trumble, B. C., Madimenos, F. C., Kaplan, H., & Gurven, M. (2014). Low mineral density of a weight-bearing bone among adult women in a high fertility population. American Journal of Physical Anthropology. 10.1002/ajpa.22681 PMC4368479 25488367

[ref56] Stieglitz, J., Trumble, B. C., Horus Study, T., Finch, C. E., Li, D., Budoff, M. J., … Gurven, M. D. (2019). Computed tomography shows high fracture prevalence among physically active forager–horticulturalists with high fertility. ELife, 8, e48607. 31418688 10.7554/eLife.48607 PMC6726459

[ref57] Studer, R. (2008). When did the Swiss get so rich? Comparing living standards in Switzerland and Europe, 1800–1913. Journal of European Economic History, 2(2), 405–452.

[ref58] Studer, R., & Schuppli, P. (2008). Deflating Swiss prices over the past five centuries. Historical Methods, 41(3), 137–156.

[ref59] Tracer, D. P. (2002). Somatic versus reproductive energy allocation in Papua New Guinea: Life history theory and public health policy. American Journal of Human Biology, 14(5), 621–626. 10.1002/ajhb.10073 12203816

[ref60] Uggla, C., & Mace, R. (2015). Local ecology influences reproductive timing in Northern Ireland independently of individual wealth. Behavioral Ecology, arv133. 10.1093/beheco/arv133

[ref61] van Buuren, S., & Groothuis-Oudshoorn, K. (2011). Multivariate imputation by chained equations. Journal of Statistical Software, 45(3), 1–67.

[ref62] Vander Werf, E. (1992). Lack's clutch size hypothesis: An examination of the evidence using meta-analysis. Ecology, 73(5), 1699–1705.

[ref63] van Noordwijk, A. J., & de Jong, G. (1986). Acquisition and allocation of resources: Their influence on variation in life history tactics. The American Naturalist, 128(1), 137–142. 10.1086/284547

[ref64] Von Rueden, C. R., & Jaeggi, A. V. (2016). Men's status and reproductive success in 33 nonindustrial societies: Effects of subsistence, marriage system, and reproductive strategy. Proceedings of the National Academy of Sciences, 113(39), 10824–10829. 10.1073/pnas.1606800113 PMC5047206 27601650

[ref65] Walker, R. S., Hill, K. R., Flinn, M. V, & Ellsworth, R. M. (2011). Evolutionary history of hunter–gatherer marriage practices. PloS One, 6(4), e19066. 10.1371/journal.pone.0019066 21556360 PMC3083418

[ref66] Zhou, X., & Reiter, J. P. (2010). A note on Bayesian inference after multiple imputation. The American Statistician, 64(1987), 159–163. 10.1198/tast.2010.09109

