# Supplementary material for: Life-history tradeoffs in a historical population (1896–1939) undergoing rapid fertility decline: Costs of reproduction?
Source: Evol Hum Sci. 2022 Feb 21;4:e7. doi: 10.1017/ehs.2022.2 (PMC7612759; doi:10.1017/ehs.2022.2)

<u>Supplementary Materials for</u>

Life-history tradeoffs in a historical population (1896-1939) undergoing rapid fertility decline: Costs of reproduction?

Adrian V. Jaeggi[1], Jordan S. Martin[1], Joël Floris[1,2], Nicole Bender[1], Martin Haeusler[1], Rebecca Sear[3], Kaspar Staub[1,4,5]

[1]Institute of Evolutionary Medicine, University of Zurich

[2]Department of History, University of Zurich

[3]London School of Hygiene and Tropical Medicine

[4] Zurich Center for Integrative Human Physiology (ZIHP), University of Zurich

[5] Institute of History, University of Bern

Contents

Table S1: Comparison of male SEP in census and the birth record data in 1920 2

Figure S1: Temporal trends in relevant socio-economic and demographic parameters 2

Figure S2: Density plot of imputed vs observed values 3

Figure S3: No effect of parity on offspring outcomes (Model 2) 3

Figure S4: Interactions between parity and maternal condition on offspring outcomes (Model 2).......... 4

Figure S5: Effects of maternal condition on parity (Model 2) 5

Figure S6: Effects of offspring sex (male vs female) on offspring condition (Model 2) 5

Table S1: Comparison of male SEP in census and the birth record data in 1920

Male SEP	Low (1,2)	Medium (3)	High (4,5,6)
1912-20 (Fathers)	19.9	42.9	37.2
Census 1920	18.8	48.1	33.2

Census 1920: Eidgenössisches Statistisches Amt (1923).

Figure S1: Temporal trends in relevant socio-economic and demographic parameters in Basel during the study period. For C), deaths <1 means infants who died before reaching their first birthday

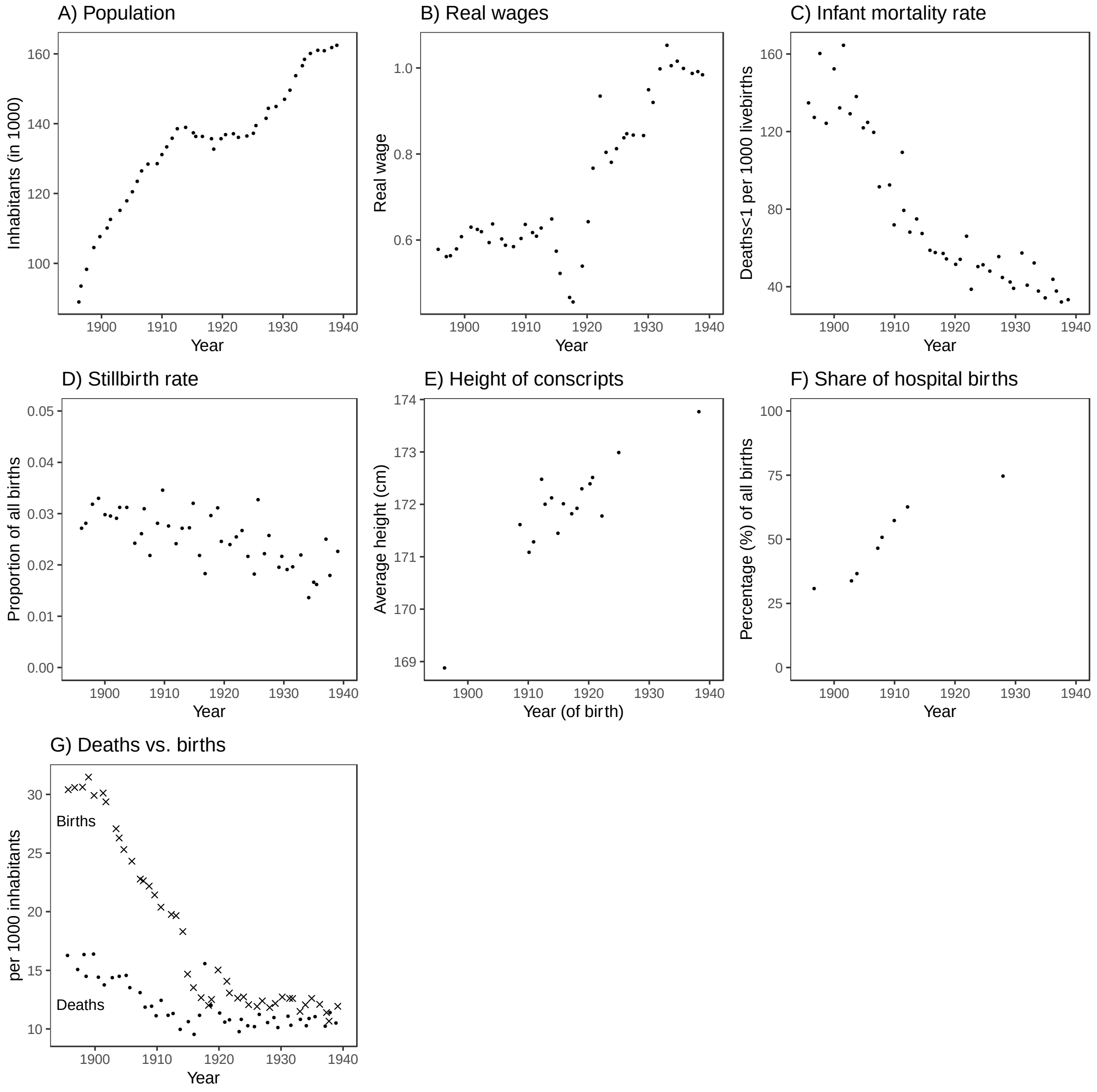

Figure S2: Density plot of imputed vs observed values. Blue lines are observed data, red lines are the 10 imputed datasets. In general, imputed data match observed ranges well, indicating that the imputation produced plausible values. The exceptions are (i) age, which is overestimated by the imputation, but only had 9 missing values, and (ii) birthweight, which was slightly underestimated by the imputation (n=90 missing values).

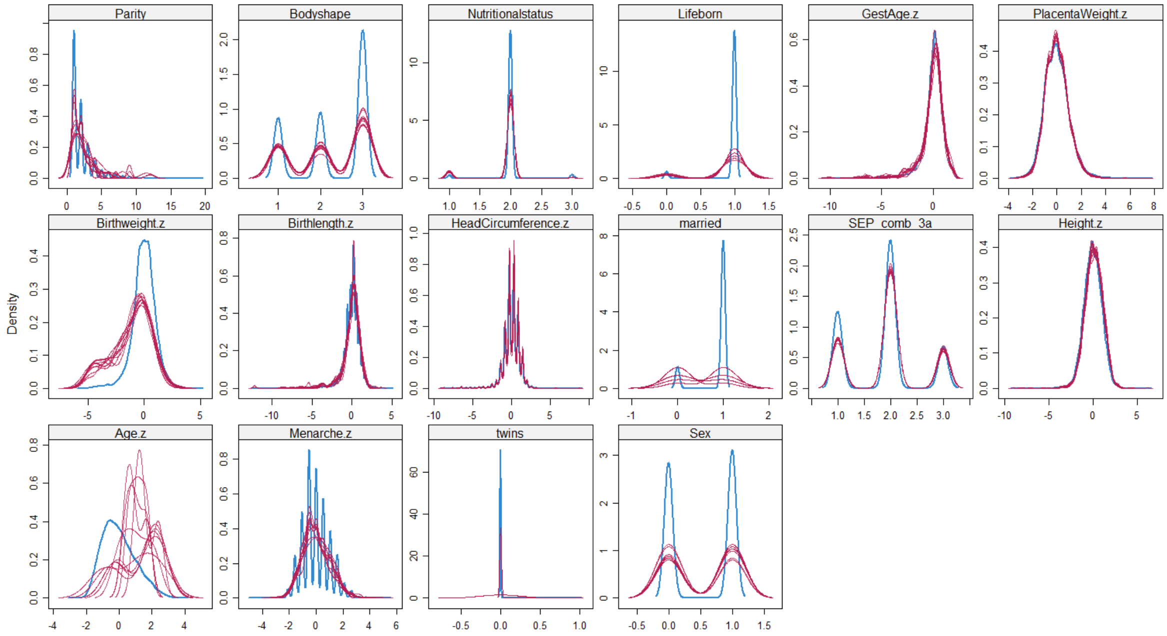

Figure S3: No effect of parity on offspring outcomes (Model 2). In contrast to Figure 1, only the main effects of parity are shown because residual correlations were not included in Model 2. Plotted are the posterior distributions with 90% credible interval, and numbers give the proportion of the posterior that supports the prediction (e.g. the proportion of the posterior <0, or $P_{<0}$). LB = Probability of live birth (expressed as an odd's ratio), GA = Gestational age, PW = Placenta weight, BW = Birth weight, BL = Birth length, HC = Head circumference

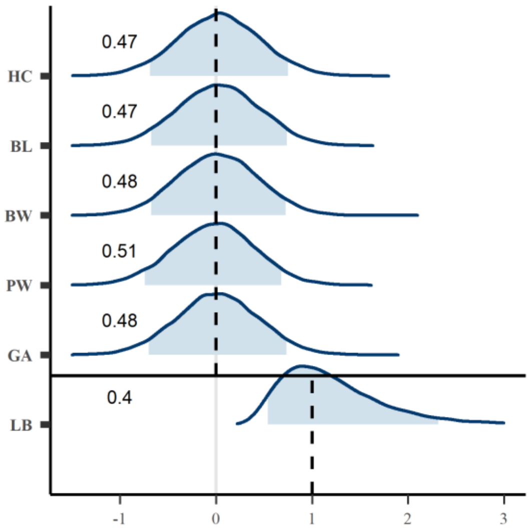

Figure S4: Interactions between parity and maternal condition on offspring outcomes (Model 2), namely a) probability of live birth, b) gestational age, c) placenta weight, d) birth weight, e) birth length and f) head circumference. Maternal condition was proxied by marital status (MS, indicating being married vs not married), height (HT), age of menarche (AM), socio-economic position (SEP), and year. Plotted are the posterior distributions with 90% credible interval, and numbers give the proportion of the posterior that supports the prediction (e.g. the proportion of the posterior <0, or $P_{<0}$). Estimates for the interaction between parity and the year spline included three parameters, which are not shown here due to lack of clear interpretability

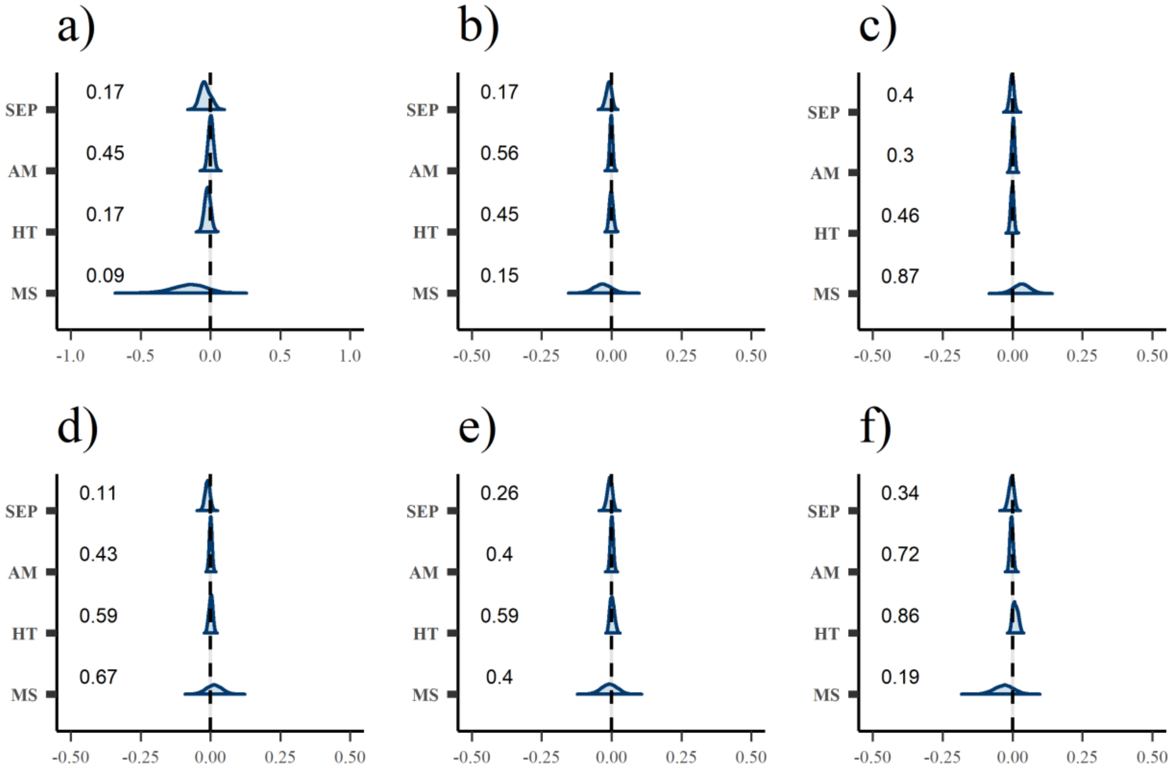

Figure S5: Effects of maternal condition on parity (Model 2). Maternal condition was proxied by marital status (MS), height (HT), age of menarche (AM), and socio-economic position (SEP). Plotted are the posterior distributions with 90% credible interval, and numbers give the proportion of the posterior that supports the prediction (e.g. the proportion of the posterior <0, or $P_{<0}$)

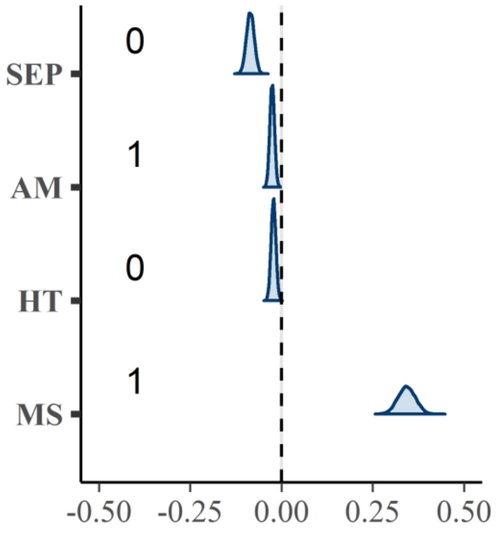

Figure S6: Effects of offspring sex (male vs female) on offspring condition (Model 2). Plotted are the posterior distributions with 90% credible interval, and numbers give the proportion of the posterior that supports the prediction (e.g. the proportion of the posterior <0, or $P_{<0}$). LB = Probability of live birth (expressed as an odd's ratio), GA = Gestational age, PW = Placenta weight, BW = Birth weight, BL = Birth length, HC = Head circumference

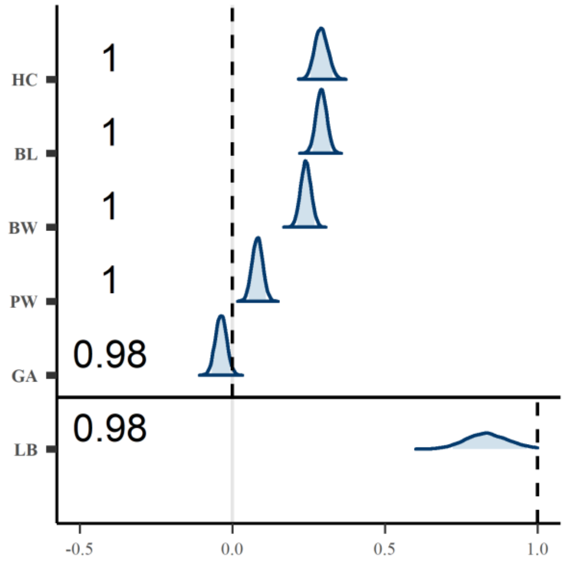

Supplement: Supplementary file 1 [file S2513843X22000020sup001.pdf]
